# Rodent models of focal cerebral ischemia: procedural pitfalls and translational problems

**DOI:** 10.1186/2040-7378-1-8

**Published:** 2009-11-25

**Authors:** Stefan Braeuninger, Christoph Kleinschnitz

**Affiliations:** 1 Department of Neurology, Julius-Maximilians-Universitaet Wuerzburg, Josef-Schneider-Str. 11, 97080 Wuerzburg, Germany

## Abstract

Rodent models of focal cerebral ischemia are essential tools in experimental stroke research. They have added tremendously to our understanding of injury mechanisms in stroke and have helped to identify potential therapeutic targets. A plethora of substances, however, in particular an overwhelming number of putative neuroprotective agents, have been shown to be effective in preclinical stroke research, but have failed in clinical trials. A lot of factors may have contributed to this failure of translation from bench to bedside. Often, deficits in the quality of experimental stroke research seem to be involved. In this article, we review the commonest rodent models of focal cerebral ischemia - middle cerebral artery occlusion, photothrombosis, and embolic stroke models - with their respective advantages and problems, and we address the issue of quality in preclinical stroke modeling as well as potential reasons for translational failure.

## Introduction

Ischemic stroke is a common disease that is one of the major causes of death and disability worldwide, as well as being a significant economic burden. Given an aging population, ischemic stroke is projected to become even more important in the future [1]. Animal stroke models have shed light on the pathophysiology of ischemic stroke [2] and numerous potential targets for stroke therapy have been identified. As of 2006, a total of 7,554 results of 1,082 putative neuroprotective interventions in experimental stroke have been published [3]. The translation of these results from bench to bedside, however, has been overall disappointing. To date, thrombolytic therapy using recombinant tissue plasminogen activator is still the only effective pharmacological treatment in acute ischemic stroke [4]. Many factors have been discussed that may have contributed to the lack of concordance of preclinical and clinical study results. Inappropriate conduct of preclinical research (especially insufficient quality) as well as suboptimal design of clinical trials (especially patient selection criteria and clinical evaluation) have been identified [5]. Lessons from translational failures led to the Stroke Therapy Academic Industry Roundtable (STAIR) conferences of academic and industrial experts from which recommendations and standard operating procedures for preclinical and clinical stroke research have been devised [6-10]. Ongoing deficits in experimental stroke research have been intensely discussed and analyzed, and additional useful recommendations for quality improvement have been published [11-15]. In this review article, the commonest rodent models of focal ischemic stroke and their respective advantages and disadvantages are presented and critically discussed with a focus on quality issues and translational problems.

## Ischemic stroke models

The classification of animal models of ischemic stroke can be based on a variety of factors such as species, occlusion mechanisms and stroke etiology, presence or absence of reperfusion (temporary/transient or permanent ischemia), the involved vascular territory and infarct distribution, or a combination of these factors.

A classification of experimental ischemic stroke according to infarct distribution seems reasonable, and with this approach global, focal and multifocal ischemia models can be differentiated [16]. Global cerebral ischemia, a model of circulatory failure, cannot be regarded as a stroke model in the strict sense and is beyond the scope of this review. Focal cerebral ischemia occurs when blood flow in a specific brain region is critically limited. It is usually the result of occlusion of a major cerebral artery, such as the middle cerebral artery. Multifocal ischemia represents a reduction of cerebral blood flow in multiple regions and has been described as patchy reduction of cerebral blood flow [16]. Multifocal cerebral ischemia can be caused by injection of embolus material into a brain-supplying artery.

The etiology of ischemia can be categorized as extravascular or intravascular [17]. Extravascular mechanisms are vessel ligation, electrocauterization, or clipping. An innovative method employing an extravascular mechanism is local application of endothelin-1 adjacent to a brain-supplying artery [18,19] or on the brain surface [20]. Intravascular mechanisms include an intravascular occluding suture in focal cerebral ischemia and injection of blood clots and other embolus material in multifocal cerebral ischemia models.

An overview of cerebral ischemia models is given in Table 1. For ethical and practical reasons, rats and - especially in transgenic studies - mice are the most widely used laboratory animals in preclinical stroke research. In the following, we focus on major models of focal stroke in these rodents.

**Table 1:** Overview of experimental stroke models

*Cerebral Ischemia*	*Etiology*	*Reperfusion (transient ischemia) or permanent ischemia*	*Examples*
			

Global (complete or incomplete) = model of circulatory arrest or severe hypotension	Intravascular	Transient or permanent	Cardiac arrest with or without cardiopulmonary resuscitation
	
	Extravascular	Transient or permanent	Cervical compression by neck cuff; ligation of several brain-supplying arteries

Focal	Intravascular	Transient or permanent	Intraluminal thread middle cerebral artery occlusion model
	
	Extravascular	Transient or permanent	Surgical middle cerebral artery occlusion models using ligation, clipping, electrocauterization etc.; endothelin-1-induced middle cerebral artery occlusion

Multifocal	Intravascular	Transient (spontaneous lysis or thrombolytic therapy possible in blood clots) or permanent	Embolization models using blood clots, microspheres or other embolus material

## Major rodent models of focal stroke

### Intraluminal thread middle cerebral artery occlusion model

The middle cerebral artery occlusion (MCAo) model using an intraluminal thread was developed by Koizumi et al. in rats [21], and has subsequently been modified [22,23] and adapted to mice [24]. Briefly, an occluding suture or monofilament is advanced via the common carotid artery into the internal carotid artery towards the junction of the anterior and middle cerebral arteries. Thus the middle cerebral artery vascular territory is subjected to ischemia. The occluding suture may be removed after various time periods, allowing for reperfusion (transient MCAo) or it may be left in place permanently (permanent MCAo).

There are several advantages of this technique: it models focal infarction in a large vascular territory and does not require craniotomy. Thus it can be regarded as relatively simple, though microsurgical skills are required.

Specific complications of the intraluminal thread MCAo model have been reported. MCAo may be complicated by subarachnoid hemorrhage in a substantial number of animals [25-28]. Other adverse events include ipsilateral retinal injury and consequent visual dysfunction [29], ischemia in the external carotid artery territory [30,31], intraluminal thrombus formation [32,33], and inadequate MCAo and premature reperfusion [26,27,34]. If the infarct involves the hypothalamus - as seems to be frequently the case in animals subjected to permanent MCAo or to longer times of transient MCAo - hyperthermia may develop as a consequence of thermal dysregulation, leading to altered stroke outcome [35-37]. Technical modifications and the use of standardized monofilaments can reduce the incidence of these complications [28,38,39]. In line with differences in the technical approach, researchers may report tremendously different incidences or may not produce certain complications in their hands at all [27]. For example, external carotid artery territory ischemia, such as temporal muscle necrosis resulting in impaired outcome (decreased body weight and delayed recovery), has been reported as frequently as in 50% of animals [30,31], but has not been confirmed by others and seems to be related to the use of electrocauterization for vessel dissection [40]. In mice [25,41-43], but also in rats [44-46], infarct volumes vary considerably between different strains and ages, and show a steep relationship to occlusion time [47]. For example, C57Bl/6 mice have significantly larger infarcts than SV129 mice in the permanent MCAo model [25,42], most likely due to the absence of one or both posterior communicating arteries in many C57Bl/6 mice [43,47,48]. These are the most widely used parent strains for the production of transgenic mice. Moreover, infarct volumes found in different murine MCAo studies using the same mouse strain and same MCAo duration can range over a fivefold difference [49]. The different absolute and relative incidences of complications, as well as minor technical differences, may contribute to the considerable variability observed with the intraluminal thread MCAo model.

### Surgical middle cerebral artery occlusion models

There are also models of MCAo that do not use an intraluminal occluding suture, but employ an extravascular mechanism (for example, ligation, clipping, or electrocauterization). These surgical MCAo models require craniotomy and incision of the dura. For example, the proximal middle cerebral artery can be identified and occluded after removal of the coronoid process of the mandible and zygoma and opening a burr hole lateral to the foramen ovale [50]. A more refined method that has been reported to cause reproducible infarctions is tandem occlusion of the distal middle cerebral and ipsilateral common carotid arteries [51]. Disadvantages of the surgical approaches, however, include technical intricacy, possible impairment of cerebral blood flow auto-regulation by damage to autonomic nerves, and craniotomy. The latter has been shown to alter brain temperature, intracranial pressure, and blood-brain barrier permeability [52,53]. For these reasons, most researchers prefer the intraluminal thread MCAo method.

### Photothrombosis model

In the photothrombosis model originally described by Watson et al. in rats [54] and later modified for mice [55], a cortical brain lesion is induced by systemic injection of a photosensitive dye, such as Rose Bengal, and subsequent focal irradiation of the skull.

Though some researchers expose the dura or brain surface prior to illumination, the method does not require craniotomy if the light penetrates the skull, and it can in this case be regarded as minimally invasive. A special advantage of photothrombosis is the high reproducibility of lesion size and location. It is even possible to determine the region of irradiation stereotactically and thus selectively induce infarcts in cortical areas representing specific functions [56]. The technique is relatively simple and allows high throughput.

The penumbra, however, an important target of many putative stroke therapeutics, is lacking in photothrombosis, where blood-brain barrier disruption and vasogenic edema develop within minutes [57,58], though modifications (ring models) seem to be able to produce a penumbra-like lesion edge [59,60]. The mechanism by which photochemically induced brain lesions develop is thought to involve vascular endothelial damage, platelet activation and subsequent thrombotic vessel occlusion [57,58]. Recently, however, we have shown that the photothrombotic lesion develops independently of the presence of functional platelets or plasmatic coagulation [61]. Thus photothrombosis does not reflect vascular-ischemic brain injury or stroke, but focal brain necrosis seems to be caused directly and irrespectively of additional endothelial damage leading to local thrombosis. These findings reveal significant differences in mechanisms of tissue injury induced by photothrombosis as compared to focal ischemia induced by MCAo, where platelet activation and the intrinsic coagulation pathway are instrumental [62,63]. When evaluating antithrombotic pharmaceuticals to limit tissue injury using the photothrombosis paradigm, one should therefore be cautious because ensuing platelet-containing thrombosis may be present, but may not be mandatory to induce tissue damage.

### Models of embolic cerebral ischemia

The first embolic stroke model was developed in dogs [64] and has subsequently been adapted to rats [65] and mice [66]. After extensive methodological modifications and novel technical developments, various experimental models of embolic cerebral ischemia exist today. Endothelial injury of large vessels, for example by irradiation of the common carotid artery after administration of a photosensitive dye [67], leads to local thrombus formation and embolic stroke in the distal vascular territory. Other stroke models use embolic materials, such as polyvinyl compounds, latex microspheres, or blood thrombi that are prepared ex vivo and injected into the common carotid artery [68-70] or into the proximal middle cerebral artery [66,71-73]. Thrombus formation can also be induced at the origin of the middle cerebral artery by local thrombin injection [74].

The main advantages of embolic stroke models are their pathophysiological relevance - embolic vessel occlusion is the most frequent cause of ischemic stroke in humans - and, if blood-borne thrombi are used, the possibility to test thrombolytic compounds. Those models not placing emboli directly in the proximal middle cerebral artery, however, often show high variability of infarct location, distribution, and size (multifocal stroke model). Spontaneous lysis of thrombi can occur and is difficult to assess. Moreover, placement or induction of emboli in the proximal middle cerebral artery is technically demanding. A common complication, especially in mice, is subarachnoid hemorrhage, reported in about 40% of mice [66]. In emboligenic endothelial lesions of the common carotid artery, lesion type (endothelial denudation versus photothrombosis) may determine the fibrin content of the thrombi, and thus their embolic potential [75].

As most experimental stroke models address the anterior circulation, models of vertebrobasilar occlusion are sparse and have been developed in larger animals such as rabbits, cats, and dogs. Recently, however, the autologeous thromboembolic stroke model [68] has been adapted to the posterior (vertebrobasilar) circulation in rats [76] allowing the study of stroke pathophysiology in the brainstem and cerebellum that may differ from the situation in the anterior circulation.

## Potential reasons for translational failures and proposed actions

### Species differences

There are, of course, significant physiological, neuroanatomical and metabolic differences between humans and small rodents, which are the most widely used experimental animals in preclinical stroke research. For example, small rodents usually require higher drug doses on a mg/kg body weight basis for a similar effect than larger mammals [77]. Thus, effective doses derived from preclinical stroke studies in small rodents cannot simply be transferred to the situation in humans, even if adjusted for body weight. Dose-response curves in laboratory animals and in humans can be helpful to address this problem.

Moreover, it should be kept in mind that mice and rats are lissencephalic. Therefore, if a drug has been effective in preclinical stroke studies in small rodents, it is recommended to reproduce the result in higher species (in particular non-human primates; for review, see Fukuda and del Zoppo [78]) prior to the initiation of clinical trials [6]. These animals are gyrencephalic and more closely resemble the situation in humans.

### Strain differences

Strain differences in mice [25,41-43] and rats [44-46] must not be underestimated and imply that results in one strain may not necessarily be reproducible in another strain. The profound genetic differences and phenotypic variance between mouse strains can explain that certain isogenic strains poorly mimic human disease or even that the effects of a targeted mutation are overshadowed, which has raised concern about the common practice of using a single mouse strain, mostly C57BL/6, to address many questions [79]. In transgenic studies in particular, not only the single gene mutation but also the genetic background must be taken into account [79]. Thus, preclinical studies of interventions in stroke should include more than one rat or mouse strain.

### Sex, age, and comorbidities

Preclinical stroke experiments are often restricted to juvenile male animals to avoid the variability caused by female hormone cycling. It was assumed that pathophysiology and response to therapy seen in male animals would also apply to the other sex. In various experimental stroke models, however, young female adult rodents had smaller infarcts than their male counterparts [80-82]. In rats, there seems to be a correlation to the estrous cycle, with high endogenous estradiol levels in proestrus being correlated with smaller infarcts than low endogenous estradiol levels in metestrus [83]. In human observational studies, premenopausal women have a smaller incidence of ischemic stroke than men; and stroke risk in both genders increases with age [84].

There is a complex and significant influence of age on stroke outcome. Most studies have reported more severe consequences of cerebral ischemia in aged as compared to young animals. For example, older rats have been shown to develop larger infarcts [85,86]. Others, however, have reported contrary results with a more favorable outcome in aged animals [45,87]. The correlation between age and brain damage may not be linear [88]. Aging is associated with microvascular changes in laboratory rodents [89], and a small decline of cerebral blood flow has been observed [90].

Rats suffering from streptozotocin-induced diabetes and spontaneously hypertensive rat strains have been shown to develop larger infarcts [45], illustrating the impact of comorbidities.

Taken together, the influence of species, strain, sex and age (and, if applicable, comorbidities) on experimental stroke must be taken into account. Whereas preclinical stroke research often uses healthy male juvenile rodents, a considerable number of stroke patients in clinical studies will be female, elderly, or suffer from comorbidities such as diabetes, hypertension and atherosclerosis.

### Stroke model

Selection of the most appropriate stroke paradigm is critical. Models requiring craniotomy, such as proximal middle cerebral artery ligation, are traumatic and do not mimic human stroke very closely. MCAo by an intraluminal thread and models using blood clot emboli are more similar to the clinical situation. Because human stroke is heterogeneous, however, there can be no single ideal stroke model. The different experimental stroke paradigms vary with respect to the underlying pathophysiological mechanisms and the size or location of the lesion. It is thus prudent to choose an approach that matches the anticipated situation in stroke patients as closely as possible. For example, thromboembolic models using blood clot emboli are helpful when the efficacy of thrombolytic drugs is to be examined. Ideally, the results should be reproducible in different models of focal ischemic stroke and a novel substance should be tested both with and without reperfusion [6].

Not only should the stroke model reflect the clinical situation, but the mechanism of action of a putative stroke drug or intervention should also be relevant for human stroke pathophysiology and, thus, has to be elucidated prior to the initiation of clinical studies. It is, for example, important that potential neuroprotective drugs are able to penetrate the blood-brain barrier. Cerebral ischemia causes disruption of the blood-brain barrier, but this occurs only several hours after stroke onset [91].

### Anesthesia

For practical and ethical reasons, experimental focal stroke has to be induced under appropriate anesthesia and analgesia. Any type of anesthesia, including inhalation anesthesia, can, however, alter stroke outcome [92]. Barbiturates have various side effects including hypothermia and may mediate neuroprotection [93,94], thus interfering with putatively neuroprotective interventions. Concern has also been raised about the use of ketamine that could overestimate the neuroprotective effect of an intervention [95]. Inhalation anesthesia is superior to intraperitoneal or intravenous administration of anesthetics concerning the control of the depth of anesthesia [96]. Even if inhalation anesthesia is used, however, animals under general anesthesia that breathe spontaneously have been shown to exhibit larger infarct volumes and a higher variability of physiological parameters than those endotracheally intubated and mechanically ventilated [96].

Potential pitfalls may thus be avoided by selection of appropriate anesthesia. Mechanical ventilation seems to reduce possible side effects of anesthesia on stroke outcome best, but may be too laborious to be applied routinely. Inhalation anesthesia should be preferred to intravenous or intraperitoneal administration of anesthetics, and it seems prudent to avoid barbiturates and ketamine.

### Flaws in basic study design

Only a minority of published experimental stroke studies have reported randomized treatment allocation and blinding of investigators [15]. In a metaepidemiologic approach examining 13 meta-analyses of experimental stroke studies, those studies with unblinded induction of ischemia reported considerably greater effects than blinded studies demonstrating bias in the design of preclinical stroke studies [97]. Thus, basic universal standards of scientific research such as blinding of investigators should be strictly followed in modeling cerebral ischemia. This should also include confirmation of results by different independent laboratories prior to the initiation of clinical studies.

### Therapeutic time window

There is a therapeutic time window in ischemic stroke [98]. It is defined as the time interval during which an intervention can lead to partial or total recovery by restoration of perfusion (reperfusion window) or by protection of penumbra tissue from necrosis (neuroprotective window). It has also been noted that a rigid and universal time window for acute stroke therapy does not exist and that the specific pathophysiological state must be taken into account to assess the individual therapeutic potential [99].

The therapeutic time window in ischemic stroke may differ critically between species, and, in addition, depends on the mechanism of drug action and is influenced by factors such as temperature or collateral circulation. The time point at which a certain drug is administered during stroke development should thus be carefully selected and has to balance maximum efficacy on the one hand and clinical applicability on the other hand. Early drug administration, or even pretreatment, is usually the most promising approach regarding efficacy [100], whereas for routine clinical practice it is desirable to develop drugs that are still effective several hours, or even days, after stroke onset. To address these problems, in-depth pharmacokinetics in experimental animals and, if possible, in humans should be determined and compared.

### Physiological variables

Physiological parameters can profoundly influence infarct development and outcome after ischemic stroke. Severe hyperglycemia has been shown to be detrimental and is associated with larger infarcts in animal stroke models [45,101]. A study using insulin after cerebral ischemia in rats found optimal blood glucose levels at approximately 6 to 7 mM and adverse effects of hypoglycemia below 3 mM [102].

Brain temperature can also have a pronounced influence in experimental stroke [103]. An interesting example to illustrate the importance of temperature is the story of MK-801, an N-methyl-D-aspartate receptor antagonist. Several studies had shown neuroprotective efficacy of N-methyl-D-aspartate receptor antagonists in experimental stroke [104-107]. For MK-801 in particular, promising results from studies in the gerbil had been published [108,109]. It was, however, observed that animals developed prolonged hypothermia following treatment with MK-801. In 1990, researchers studied the effects of (a) MK-801 administration, (b) MK-801 administration in animals kept normothermic, and (c) hypothermia without drug administration [110,111]. Significant and similar effects were only found in (a) and (c). Thus it was demonstrated that the beneficial effects of MK-801 in experimental stroke, previously thought to be mediated by a specific mechanism involving N-methyl-D-aspartate receptor antagonism, could in fact be explained by hypothermia. Hypothermia can not only reduce infarct size, but may also alter infarct distribution [112].

Since arterial blood pressure determines the size of the area where cerebral blood flow is reduced, induced hypotension in animals subjected to focal cerebral ischemia may cause larger infarcts [113]. In line, induced hypertension during [114] and after [115] an episode of focal cerebral ischemia has been shown to ameliorate infarct size. In the rat intraluminal thread MCAo model, dependency of damage on blood pressure and on MCAo duration has been demonstrated [116]. Of course, the level of arterial blood gases (pO2, pCO2) and pH [96,117,118] can also influence the development of experimental stroke, but though these parameters are often monitored, it seems that their impact has not been studied systematically and extensively in rodent focal cerebral ischemia models.

Given the impact of physiological variables such as body and in particular brain temperature, blood pressure, pH, blood gases, and glucose levels on stroke initiation, development and outcome, it can be helpful to monitor these parameters. To avoid prolonged operation times, however, recording of all relevant physiological variables in each individual animal may not be feasible. Except for experiments modeling comorbidities such as hypertension or diabetes, physiological variables should usually be kept within normal limits. By any means, special care must be taken to prevent hypothermia during and after surgery. For this purpose, heating pads, heated water baths, closed chambers with heating fans, and heating lamps are available and should be used routinely [119].

### Outcome analysis

The outcome analysis should include infarct size, histology, mortality rate and frequency of complications such as subarachnoid hemorrhage, and functional (behavioral, motor and cognitive) scores. Since morphologically intact tissue does not always imply intact function and does not exclude delayed lesion development [120,121], endpoints should not be restricted to infarct size, but functional and neurological evaluation should also be performed. Moreover, histological, biochemical, and molecular endpoints should be studied to address the multiple spatially and temporally distributed molecular and cellular changes in the infarct core and penumbra that occur after focal cerebral ischemia [122]. Especially if tissue size varies (for example, due to extensive brain edema), infarct volumes or long-term atrophy should not be determined as absolute values (mm^3^), but as relative measures (percentage of the contralateral hemisphere) [116]. The extent of the lesion and functional deficits may change over prolonged time periods [123], whereas the duration of preclinical experiments is usually limited. It can thus be helpful to extend the time frame and study long-term outcome. An elegant way to study the kinetics of infarct development and to scan for possible hemorrhagic transformation, for example after therapeutic interventions, is the serial use of magnetic resonance imaging in individual animals [62,63]. Magnetic resonance imaging in laboratory animals subjected to experimental stroke can even detect diffusion/perfusion mismatch [124] or provide information on the metabolic state [125]. Mortality rate and frequency of complications should not only be recorded, but information should also be given on excluded animals. The exclusion of certain animals from the outcome analysis may completely alter results [11]. Deletion of outlier data can be reasonable, but exclusion and inclusion criteria have to be defined a priori.

### Publication of preclinical stroke studies

It has been demonstrated that publication bias is evident in clinical stroke trials [126]. To address this problem, it has been suggested that all clinical trials should be registered and published irrespective of outcome. For a candidate neuroprotective drug, nicotinamide, it has been shown that publication bias also plays a role in preclinical stroke research: Those investigations published in abstract form showed a smaller effect of nicotinamide compared to those published as full articles [127]. This illustrates that results of preclinical stroke trials, whether positive or negative, should be completely reported and presented to the scientific community. If electronic journals are used, journal space must not be a limiting factor precluding the publication of negative results [128]. To reduce the risk of translational failure, all relevant information on preclinical studies should be available in peer-reviewed print or electronic journals when planning the design of clinical trials.

### Coordination of preclinical and clinical research

Evidently, animal models of human diseases cannot be more than an approximation to the situation in humans and are always limited by nature. In experimental stroke models, variables that influence the development of the ischemic brain lesion are controlled to ensure homogeneity, reliability and reproducibility of the results. These factors include age and sex of the animals studied, physiological parameters, and the experimental protocol. In contrast, patient selection criteria in clinical studies cannot achieve similar homogeneity as in preclinical experiments. Perhaps extreme homogeneity would not even be desirable, given that study results must be meaningful for everyday clinical practice. Study patients may differ in type, location and development of the ischemic stroke lesion, age, sex, comedication, comorbidities, physiological parameters, and time of application of the study medication. To account for the heterogeneity, large numbers of patients have to be included in clinical trials [129].

Although these problems cannot be completely circumvented, they may be ameliorated by appropriate coordination of preclinical and clinical research. The design of basic investigations should consider future translation to the clinic from the very beginning. Conversely, if experimental treatment has been effective, clinical trials should adhere as closely as possible to the conditions under which the preclinical data were collected. There is evidence that this has been neglected in the past: of more than 1,000 potential neuroprotective drugs studied in animals as of 2006, at least 114 had been tested in acute stroke patients, yet, interestingly, those drugs studied in humans were not those that had shown superior efficacy in animal studies [3].

Heterogeneity of patients in clinical stroke studies can be taken into account at the level of preclinical stroke research. Protocols solely using juvenile male animals may produce more homogeneous and robust results. Prior to the initiation of clinical studies, however, it seems to be prudent to perform additional experiments in female and aged animals or in strains bearing vascular risk factors such as obesity, hypertension, or diabetes. Indeed, it has been demonstrated that the use of healthy animals instead of animals with comorbidities overstates the effects of interventions in experimental stroke research [97].

Considerations and proposed actions for the design of preclinical stroke studies from this review are summarized in Appendix 1. They overlap and partly extend the STAIR recommendations for preclinical stroke drug development [6,13,14] and are intended to help avoiding procedural pitfalls and translational problems. It seems unlikely that there is one "ideal" preclinical stroke study evaluating a stroke drug or intervention comprehensively. Usually, several different experiments will address different issues.

## Summary

The presence of intact blood-perfused and then temporarily or permanently occluded vasculature is essential for the study of ischemic stroke. Research in healthy human subjects or in stroke patients is very limited. Moreover human stroke may be very heterogeneous; there may be many confounding variables, and it may not be possible to assess physiological and outcome parameters rigorously enough. Therefore, animal models, and in particular rodent models, play a pivotal role in stroke research in addition to in vitro techniques such as brain slices, tissue and cell culture. Rodent models of focal cerebral ischemia have added tremendously to our understanding of the etiology and pathophysiology of ischemic stroke. The translation of therapeutic approaches from the animal model to patient care, however, has been less fruitful. With the notable exception of intravenous thrombolysis with tissue plasminogen activator [4,130], the overwhelming majority of drugs showing promise in preclinical research have failed in subsequent clinical trials. All available stroke models have inevitable shortcomings and problems. Lessons to improve preclinical research methodology and to better coordinate preclinical and clinical stroke research have been derived and should be followed to reduce the risk of future translational failures.

## Competing interests

The authors declare that they have no competing interests.

## Authors' contributions

SB and CK contributed equally.

## Appendix 1 - Synopsis of considerations and proposed actions to improve the design of preclinical stroke studies

1) Select species. Small rodents such as mice and rats should be used initially (advantages: ethics, costs, practicability). After promising results in other species, studies in non-human primates (advantages: gyrencephalic, closer to human situation) should be considered.

2) Select strain. Ideally, results (both positive and negative) should be confirmed in other strains.

3) Consider animal studies in juvenile male animals (advantages: homogeneity, reproducibility). Perform additional studies in female, aged or comorbid animals (advantage: closer to clinical situation).

4) Select stroke model. The selected stroke model should be pathophysiologically relevant for the anticipated clinical situation and match the putative mechanism of the drug or intervention studied (for example, embolic stroke model using blood-borne thrombi for evaluation of thrombolytics). Stroke models with reperfusion (advantage: more common in human stroke) should be preferred over permanent ischemia models, or, ideally, a putative drug or intervention should be evaluated in both transient and permanent stroke models.

5) Select anesthetic and anesthesia method. Inhalation anesthesia should be preferred to intravenous or intraperitoneal drug administration. The potential interference of anesthetics with the stroke drug or intervention studied should be considered. In particular, barbiturates should be avoided.

6) Include basic scientific standards such as randomization and blinding of investigators; define inclusion (for example, postoperative neurological score indicating clinical deficit) and exclusion criteria a priori.

7) Determine pharmacokinetics and dose-response curves. From these, information on dosage and therapeutic time window is derived.

8) Monitor and control relevant physiological parameters, at least account for the profound effects of brain temperature.

9) Consider infarct size, functional/neurological scores, histological, biochemical and molecular evaluation for outcome analysis. The outcome analysis should at least not be restricted to only infarct size or histology.

10) Ideally, study both short-term and long-term outcome. Serial magnetic resonance imaging examinations may be helpful.

11) Elucidate mechanism of stroke drug or intervention studied. The mechanism should be relevant for human stroke.

12) Consider combination therapy (for example, putative neuroprotective drug plus thrombolysis).

13) Publish both positive and negative results.

14) Confirm results in other laboratories.

15) Coordinate preclinical and clinical research. The anticipated clinical situation should be considered in preclinical study design; clinical studies should adhere as closely as possible to the conditions in preclinical studies that showed efficacy.
